# The Effect of Change of Working Schedule on Health Behaviors: Evidence from the Korea Labor and Income Panel Study (2005–2019)

**DOI:** 10.3390/jcm11061725

**Published:** 2022-03-20

**Authors:** Saemi Jung, Seung-Yeon Lee, Wanhyung Lee

**Affiliations:** 1 Department of Occupational and Environmental Medicine, Pusan National University Yangsan Hospital, Yangsan 50612, Korea; saemi.bright@gmail.com; 2 Department of Family Medicine, International Healthcare Center, Seoul National University Bundang Hospital, Seongnam 13620, Korea; leesy8503@snubh.org; 3 Department of Occupational and Environmental Medicine, Gil Medical Center, Gachon University College of Medicine, Incheon 21565, Korea

**Keywords:** shift work, work schedule, health behavior, smoking, alcohol drinking, occupation

## Abstract

This study investigated whether changes in work schedule are associated with health behavior changes. We used data from the Korea Labor and Income Panel Survey from 2005 to 2019. A generalized estimating equation model was used to assess the association between changes of work schedules (day–day, day–shift, shift–day, and shift–shift) and health behaviors. Odds ratios (ORs) with 95% confidence intervals (CIs) were calculated after adjusting for general and socioeconomic characteristics. Fixed daytime work was observed for 25,716 person-years, and fixed shift work was observed for 2370 person-years out of the total 4046 participants during a 14 year period. Workers who changed their work schedule from fixed daytime to shift work and from shift to fixed daytime work contributed to 670 and 739 person-years, respectively. Considering continuous fixed daytime workers as a reference group, continuous exposure to shift work (aOR 1.11, CI 1.01–1.26) and changes from fixed daytime to shift work (aOR 1.18, CI 1.05–1.44) were significantly associated with an increased risk of changing either smoking or drinking behavior to unhealthy patterns. The results of our study suggest that workers who work irregular shift times, in contrast to those with more standard, regular work schedules, are at a higher risk of changing smoking and/or drinking behavior to unhealthy patterns.

## 1. Introduction

Globally, 10–40% of workers are engaged in shift work, and the number of shift workers is increasing in many countries [1]. With a consistent increase in shift work over the past few decades, the impact of shift work on health has been of great interest. Growing evidence indicates that shift work increases the risk of cardiovascular disease [2], obesity [3], type 2 diabetes [4], and other metabolic disturbances [5] as well as mental disorders [6]. Such illnesses have often been cited as being the result of poor lifestyle behavioral choices [7,8], and thus are known as lifestyle diseases. Thus, a better understanding of the relationships between shift work and health behaviors is important to provide opportunities for improving the health of shift workers and to prevent future adverse health effects.

As shift workers work outside normal daylight hours, it has been postulated that negative health behaviors in shift workers are probably related to the misalignment between intrinsic circadian and social rhythms [9]. Circadian misalignment can lead to sleep disturbances, with serious consequences for subsequent morbidities [10]. In addition, shift workers have greater difficulties combining their work and social lives, which may result in psychosocial stress [11]. To cope with such physiological and psychosocial stress, shift workers might use substances such as alcohol and tobacco.

Several studies have shown that smoking is more prevalent among shift workers than among daytime workers. In a prospective cohort study of 233,869 person-years conducted from 1988 to 1990 in Japan, higher prevalence of smoking was reported among rotating shift-workers than day workers [12]. In another prospective study for 21-year follow-up among young Finnish adults, shift work was related to higher level of smoking regardless of gender [13]. Even though a short prospective study did not show the association between change of smoking habit and shift work schedule [14] and another 6-year cohort study with Norwegian nurses did not show a significant difference in prevalence of smoking according to changes in work shift schedules [15], recent systematic reviews showed that majority of studies, including cohort studies, showed that shift work was associated with an increased risk of current and future smoking status [15,16].

Although there were some studies with inconsistent results on the increased risk of alcohol consumption among shift workers [15,17], the majority of studies showed that shift work was associated with a higher risk of heavy drinking, compared with day work [18,19]. Similarly, a positive association between shift work and alcohol consumption was reported in a recent systematic review of 14 studies [20]. 

However, the question of causality between shift work and health behaviors could not be confirmed because most of the studies were cross-sectional or limited by a short follow-up. To further clarify this relationship, more long-term detailed studies are needed. Therefore, this study aimed to investigate the longitudinal association between changes in work schedule and changes in smoking and drinking status using 14 year intra-individual follow-up data. 

## 2. Materials and Methods

### 2.1. Data and Study Participants

We used a dataset from the Korea Labor and Income Panel Survey (KLIPS) conducted by the Korea Labor Institute [21]. The KLIPS is a longitudinal survey of a representative sample of Korean households and individuals living in urban areas. The KLIPS was designed to track the economic activities of individuals and households, with a particular focus on understanding changes in the labor market and the effectiveness of policies for workers. This includes family economic status, housing status, incomes, expenditures, individual economic activities, education, occupational characteristics, job satisfaction, and lifestyle. The first wave of the KLIPS began in 1998, and the latest survey (the 23rd wave) ended in 2020. The sampling method used at baseline was a two-stage stratified clustering. A total of 5000 households and individuals (about 13,000 men and women aged 15 and over) in urban areas were selected, excluding those in rural areas. However, considering the relatively low population ratio in rural areas of Korea (18.0% of the total population as per the 2010 Population Census), this study holds significant value as the representative data of Korea. To give an equal probability of being sampled, weights were assigned to each respondent, allowing the results to represent the Korean population. This weighting method ensures unbiased point estimates of population parameters for the entire population and its subsets.

**Figure 1:**
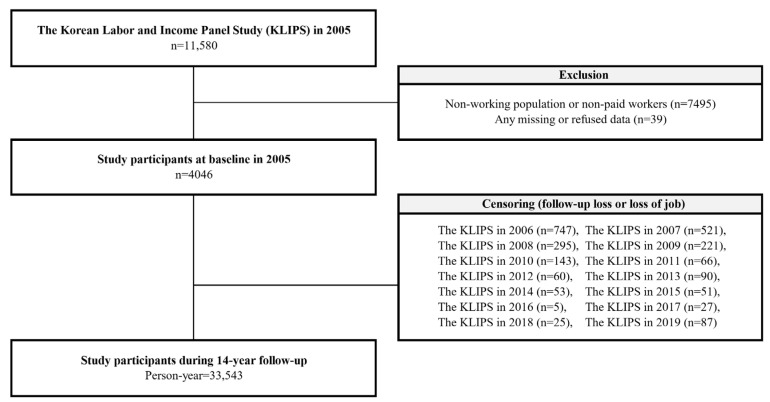
Schematic diagram depicting the study population.

The current study used the KLIPS dataset from 2005 to 2019, including information about the work schedule and health behavior that included all the information needed for the study. We excluded participants who were non-working or non-paid workers (*n* = 7495) or any missing or refused data (*n* = 39) from 11,580 subjects at baseline in 2005. A total of 4046 participants were selected at baseline, and the cohort was followed in the same way, using exactly the same questions and data collection methods until 2019. During the 14 year follow-up period, 33,543 person-years were demonstrated. Figure 1 shows a detailed schematic representation of the study population.

### 2.2. Key Variables

**Figure 2:**
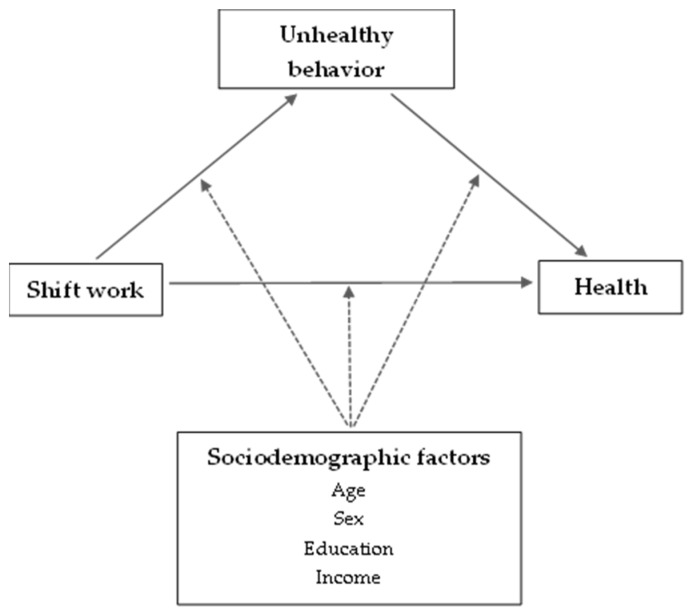
Shift work and health behavior: a conceptual framework.

Figure 2 shows a conceptual framework for the relationship between shift work, health behavior, and health. As indicated in the framework, this study presumed that shift work is associated with negative health behaviors (i.e., smoking and drinking). To address some of the existing gaps in evidence from earlier studies, we followed intra-individual changes in work schedules and health behaviors over 14 years. Age, sex, education, and income were considered as potential mediating factors.

To investigate the intra-individual changes in work schedules, we analyzed the data after grouping workers based on their work schedules at baseline and at the end of the follow-up period of 14 years. First, work schedules were defined as binominals using questionnaire responses regarding the working time schedule, fixed daytime work and shift work. Subsequently, participants were classified into the following four groups according to the changes in work schedules: (i) day–day workers, those who continued fixed daytime work; (ii) day–shift workers, those who switched from fixed daytime to shift work; (iii) shift–day workers, those who switched from shift to fixed daytime work; and (iv) shift–shift workers, those who continued shift work. 

Changes in smoking and/or drinking status (i.e., from never or past smoking to current smoking; from never or social drinking to binge drinking) were the primary outcomes of the current study. Smokers were categorized into two groups: (i) current smokers, those who reported smoking at the time they participated in a survey; and (ii) non-smokers or past smokers, those who do not currently smoke and who previously smoked or never smoked. Drinkers were also categorized into two groups. Binge drinkers were defined as those who consume alcohol more than three times per week, and the rest were defined as never or social drinkers. Current smokers and binge drinkers were classified as having unhealthy patterns of smoking and drinking behavior. The amount of alcohol consumed could not be applied due to the lack of information in the KLIPS.

### 2.3. Covariates

Age, sex, education level, and income level were used as socioeconomic characteristics. Education level was categorized as middle-school graduate or lower, high-school graduate, or college or higher. Income level was categorized based on monthly wage income: under $1500, $1500 to $2000, $2000 to $2500, and over $2500. The current study used a modified occupational classification with four categories, based on a previous study: white-collar, pink-collar, green-collar, and blue-collar workers [22]. White-collar workers included legislators, senior officials, managers, professionals, technicians, associate professionals, and clerical support workers. Pink-collar workers included sales and service professionals. Green-collar workers included skilled agriculture, forestry, and fishery workers. Finally, blue-collar workers consisted of craft and related trade workers, plant and machine operators, assemblers, and elementary occupations. Those with armed forces occupations were excluded from the study. The self-rated health status was classified as good, moderate, or poor.

### 2.4. Statistical Analysis

The prevalence of socioeconomic, occupational, and lifestyle characteristics was compared with work schedule using the chi-square test. The generalized estimating equation was used to analyze the association between changes in work schedule and changes in smoking and/or drinking status after adjusting for age in the observation year, sex, education level, occupational classification, monthly wage income level, and self-rated health status. Odds ratios (ORs) with 95% confidence intervals (CIs) were calculated. All analyses were performed using SAS, version 9.4 (SAS Institute, Cary, NC, USA). For all statistical calculations, a two-tailed *p*-value of < 0.05 was considered statistically significant. 

## 3. Results

The baseline characteristics of the study participants (4046), consisting of 422 shift workers (10.4%) and 3624 non-shift workers (89.6%), are presented in Table 1. Shift work was most common in the youngest age group (15–20 years; 23.4%). Shift workers were more often men (12.3%) and less educated (middle-school graduates, 11.1%; high-school graduates, 14.8%) compared to non-shift workers. Among the shift workers, 16.4% were blue-collar workers, 13.6% were pink-collar workers, and 4.1% were white-collar workers. None of the shift workers were green-collar workers. Shift workers did not differ from non-shift workers in their monthly wage income, self-rated health status, or smoking and drinking status.

Table 2 shows the prevalence of smoking and drinking status changes according to the changes in work schedules during the 14 year follow-up period. Of the study participants, there were 25,716 person-years for day–day workers, 670 person-years for day–shift workers, 739 person-years for shift–day workers, and 2370 person-years for shift–shift workers. Changes in smoking status from past or never smokers to current smokers were most common among shift–day workers (6.5%), followed by day–shift workers (6.4%), shift–shift workers (6.2%), and day–day workers (5.2%). Changes in drinking status from never or social drinkers to binge drinkers were most common among day–shift workers (14.5%), followed by shift–shift workers (12.9%), day–day workers (12.4%), and shift–day workers (12.0%). Changes in either smoking or drinking status were most common in day–shift workers (18.5%), followed by shift–shift workers (17.7%), shift–day workers (16.5%), and day–day workers (16.0%).

Table 3 shows the longitudinal association between changes in work schedule and changes in smoking and drinking status using the generalized estimating equation model. Considering day–day workers as the reference group, day–shift workers had a 1.19-fold (adjusted odds ratio (aOR) = 1.19, 95% confidence interval (CI) = 1.02–1.49) higher risk of drinking status change from never or social drinking to binge drinking. The aOR for smoking status change from never or past smoking to current smoking in day–shift workers was 1.20 (aOR = 1.20, CI = 0.87–1.65), but the difference was not statistically significant. Shift–shift workers had increased aORs for smoking status change (aOR = 1.05, CI = 0.87–1.26) and drinking status change (aOR = 1.12, CI = 0.98–1.27) but they did not reach statistical significance. The risk of changing either smoking or drinking status increased 1.18-fold (aOR = 1.18, CI = 1.05–1.44) and 1.11-fold (aOR = 1.11, CI = 1.01–1.26) in day–shift workers and shift–shift workers, respectively. Among shift–day workers, there were no significant differences in the risk of changing smoking and/or drinking status compared to day–day workers.

The effects of other covariates (i.e., age in observation year, sex, education level, occupational classification, monthly wage income level, and self-rated health status) on changes in smoking and drinking status are shown in Supplementary Table S1.

## 4. Discussion

In the current study, we aimed to investigate the effect of shift work on health behaviors. Many studies have investigated the relationship between shift work and health behaviors, but most of them had cross-sectional designs. More long-term detailed studies are needed to clarify this relationship further. We analyzed the data after grouping workers based on their work schedules at baseline and at the end of the 14 year follow-up period. We found that continuous exposure to shift work and changes from fixed daytime to shift work were significantly associated with a modestly increased risk of changing either smoking or drinking behavior to unhealthy patterns. This effect was more prominent in those who switched from fixed daytime to shift work. However, there were no significant changes in smoking or drinking behaviors among those who switched from shift to fixed daytime work. This suggests that workers who work irregular shift times, in contrast to those with more standard, regular work schedules, are at a higher risk of engaging in unhealthy behaviors, such as smoking and drinking alcohol.

These results are consistent with those of other studies. The risk of high-density drinking increased by 28% among night shift workers compared with daytime shift workers [19]. Fujino et al. found that shift workers had a higher current smoking rate [12]. Moreover, Amelsvort et al. showed that the proportion of smokers and the number of cigarettes smoked per day increased more for shift workers than for daytime workers in a 1-year follow-up study [23]. They also reported that it was much easier for shift workers to start smoking than daytime workers in another prospective study [24].

However, when a separate investigation was conducted on changes in drinking and smoking behaviors, no significant differences were found according to changes in work schedule. One of the possible reasons for this finding is the potential selection bias that healthier people are more likely to be selected for shift work, while unhealthy people are less likely to be selected for shift work. In general, healthy individuals have more intentions to engage in positive health behaviors. This ‘healthy shift worker effect’ can dilute the results on the association between shift work and health behaviors. Another reason may be the global trend toward reduced smoking. In addition, shift workers may have fewer opportunities to enjoy drinking than daytime workers because they have to adjust their lifestyle according to their sleep–wake cycle. 

Shift workers are exposed to high levels of job stress. Several studies have shown that stress is associated with substance use, smoking, and drinking. The mechanism of the effect of stress on smoking or drinking across the initiation, maintenance, and relapse stages can be explained mainly on two grounds [25]. First, smoking and alcohol consumption can be used to relieve anxiety and depression caused by stress. Stress activates dopaminergic neurotransmission in the mesolimbic regions of the brain, thereby enhancing the reinforcement of drug craving and abuse [26]. Nicotine and ethanol share the same neurobiological mechanisms as stress in the regulation and reward pathways. Nicotine activates both the hypothalamic–pituitary–adrenal axis and autonomic nervous system, resulting in an increased secretion of catecholamines such as cortisol and norepinephrine [27], which reinforces the reward pathway, increasing the risk of intensive use and relapse of smoking [28]. In addition, as ethanol reduces the activity of gamma-aminobutyric acid (GABA) release, resulting in the disinhibition of dopamine-releasing neurons in the mesolimbic system, the likelihood of ethanol-seeking behavior increases in response to the stress that activates the dopamine-releasing neurons [29,30]. Second, job stress can reduce self-control. Since self-control is a limited resource, the more stress people experience, the less self-control there is to not smoke or drink. Reduced self-control makes it difficult to maintain cessation of smoking or drinking and may induce relapse of smoking or drinking [31,32]. 

Another suggestion to explain how shift work promotes unhealthy behavioral choices is sleep loss. Shift work, a set of nontraditional work schedules outside normal daylight hours, for example, evening, night, and early morning shifts, can disrupt a person’s sleep–wake cycle through a mismatch of circadian rhythms [33]. The circadian rhythm is a 24 h body clock cycling between alertness and sleepiness that responds to light changes in the environment. During the day, light exposure causes the body to send signals that generate alertness, which helps to keep us awake. As it gets dark, the body initiates the production of melatonin, a hormone that promotes sleep [34]. Any situation that desynchronizes the internal sleep–wake cycle and the social light–dark cycle (e.g., irregular light exposure during shifts) can cause sleep difficulties as well as problems in maintaining alertness. Indeed, several studies have shown that shift workers are at a higher risk of sleep disorders than non-shift workers [35,36]. Sleep disturbances are likely to be associated with unhealthy lifestyle behaviors such as smoking and alcohol consumption [37]. Nicotine exposure from smoking induces mild euphoria, increases alertness, enhances concentration, and reduces stress [38]. These psychoactive effects of nicotine may explain the correlation between smoking behaviors and sleep problems in shift workers. According to a Japanese survey on smoking behaviors among nurses, the two major reasons for smoking were mood changes and sleepiness reduction [39]. This suggests that shift workers are prone to smoking to relieve stress and to counter sleepiness and tiredness. Sleep problems may also lead to heavy drinking because alcohol is commonly used as a sleep aid [40]. A large-scale global cross-sectional survey conducted in 10 countries reported that approximately 20% of participants who thought they did not sleep well drank alcohol [41]. People who drink often can become tolerant to the sedative effects of alcohol, which in turn may lead to the consumption of more alcohol [42]. Accordingly, shift workers who suffer from sleep problems and use alcohol to help them sleep may be at risk of heavy drinking. Shift workers may be more prone to smoking to overcome sleepiness and fatigue. They may also attempt to relieve stress by smoking and consuming alcohol. Such unhealthy behaviors, including smoking and alcohol consumption, influence the future incidence of various chronic diseases [43]. In addition to lifestyle behaviors, circadian rhythm misalignment causes problems at the molecular and hormonal level. This increases the release of inflammatory markers that are positively associated with a higher incidence of cardiovascular disease, sleep disturbances, metabolic syndrome, obesity, and cancer [44]. Therefore, we should understand the fundamental situations that can cause unhealthy behaviors among shift workers and try to prevent future adverse health effects. 

To the best of our knowledge, this is the first study in Korea to investigate the association between changes in work schedules and lifestyle behaviors using longitudinal data. Considering that individual behaviors affect a person’s risk of developing a range of diseases, potential long-term changes in lifestyle behaviors could be of interest in clinical practice. The longitudinal design of our study with a 14 year follow-up period enabled us to investigate within-person changes in smoking and drinking behaviors in relation to changes in work schedules from a long-term perspective. Furthermore, our findings can be generalized to the wider population of South Korea because a large, nationally representative dataset from the KLIPS was used. 

However, some limitations of this study must be considered when interpreting the results. First, our study relied on self-reported data, which may have introduced some response bias. Second, we could not confirm the causal effect of work schedule changes on health behavior changes because of the observational nature of the study. Third, due to a lack of data, we were unable to adopt all co-variables in our analysis, such as the type of shifts, working hours, workload, workplace stress, and medical histories. Fourth, there is a lack of history of work schedules between the baseline and the endpoint. Therefore, a potential ‘healthy shift worker effect’ (i.e., that healthier people are more likely to be selected for shift work, while unhealthy people are not selected for shift work) could have diluted the results. Fifth, our study only focused on evaluating the association between changes in work schedule and changes in health behavior. We discovered that work schedule changes to nonstandard, irregular shift times can lead to unhealthy lifestyle choices, but we did not further investigate their clinical impact on health. Future studies are required to show whether changes in behavioral patterns affect the distribution and risk of disease and other health-related outcomes in the population. Sixth, our data only reflect the experiences of those in South Korea, which may not be applicable to other countries or cultures. 

## 5. Conclusions

This study raises the possibility that changes in work schedules could trigger unhealthy lifestyle behaviors, which is an important issue for workers’ health. In particular, workers who changed their work schedule from fixed daytime to shift work were vulnerable to adopting unhealthy lifestyle behaviors, such as smoking and drinking alcohol. A further study with a greater focus on working schedule changes and their effects on workers’ health is suggested.

## Figures and Tables

**Table 1:** General characteristics of the study participants at baseline according to shift work in 2005.

	Shift Work, *n* (%)		*p*-Value *
	No	Yes	
No. of participants	3624 (89.6)	422 (10.4)	
Age (years)			0.0002
15–20	36 (76.6)	11 (23.4)	
21–40	2078 (91.1)	204 (8.9)	
41–60	1355 (89.2)	164 (10.8)	
>60	155 (78.3)	43 (21.7)	
Sex			<0.0001
Male	2164 (87.7)	304 (12.3)	
Female	1460 (92.5)	118 (7.5)	
Education level			<0.0001
Middle school	713 (88.9)	89 (11.1)	
High School	1205 (85.2)	209 (14.8)	
College or higher	1706 (93.2)	124 (6.8)	
Occupational classification			<0.0001
White-collar	1743 (95.9)	74 (4.1)	
Pink-collar	478 (86.4)	75 (13.6)	
Green-collar	10 (100.0)	0 (0.0)	
Blue-collar	1393 (83.6)	273 (16.4)	
Monthly wage income ($, USD)			0.0911
<1500	929 (87.8)	129 (12.2)	
1500–2000	899 (91.2)	98 (9.8)	
2000–2500	668 (91.4)	63 (8.6)	
>2500	1128 (89.5)	132 (10.5)	
Self-rated health status			0.6508
Good	2174 (89.4)	258 (10.6)	
Moderate	1204 (90.1)	132 (9.9)	
Bad	246 (88.5)	32 (11.5)	
Smoking status			0.4557
Never or past	2321 (89.8)	263 (10.2)	
Current	1303 (89.1)	159 (10.9)	
Drinking status			0.0696
Never or social	2310 (90.2)	250 (9.8)	
Binge	1314 (88.4)	172 (11.6)	

* Comparison using chi-square test.

**Table 2:** Changes in smoking and drinking status according to changes in shift work schedule during the 14 year follow-up period (person-years, %).

Work Schedule Change Each Year	Total (Person-Years)	Healthy to Unhealthy Behavior	Never or Past to Current Smoking	Never or Social to Binge Drinking
Day–Day workers	25,716	4117 (16.0)	1338 (5.2)	3181 (12.4)
Day–Shift workers	670	124 (18.5)	43 (6.4)	97 (14.5)
Shift–Day workers	739	122 (16.5)	48 (6.5)	89 (12.0)
Shift–Shift workers	2370	419 (17.7)	148 (6.2)	307 (12.9)

‘Healthy to unhealthy behavior’ indicates changes from ‘Never or past to current smoking’ or ‘Never or social to binge drinking’.

**Table 3:** Results of a generalized estimating equation analyzing the effect of changes in shift work schedule on changes in smoking and drinking status during the 14 year follow-up period.

Work Schedule Change Each Year	Healthy to Unhealthy Behavior	Never or Past to Current Smoking	Never or Social to Binge Drinking
	OR (95% CI)	OR (95% CI)	OR (95% CI)
Day–Day workers	1.00	1.00	1.00
Day–Shift workers	**1.18 (1.05–1.44)**	1.20 (0.87–1.65)	**1.19 (1.02–1.49)**
Shift–Day workers	1.01 (0.83–1.23)	1.21 (0.89–1.63)	0.95 (0.76–1.20)
Shift–Shift workers	**1.11 (1.01–1.26)**	1.05 (0.87–1.26)	1.12 (0.98–1.27)

Bold indicates statistical significance. Risk expressed as odds ratio (95% confidence interval). All results were adjusted for age in observation year, sex, education level, occupational classification, monthly wage income level, and self-rated health status. ‘Healthy to unhealthy behavior’ indicates changes from ‘Never or past to current smoking’ or ‘Never or social to binge drinking’.

## Data Availability

Data are available at: https://www.kli.re.kr/klips_eng/index.do (accessed on 2 February 2022).

## Supplementary Materials

The following supporting information can be downloaded at: https://www.mdpi.com/article/10.3390/jcm11061725/s1. Table S1. Results of a generalized estimating equation analyzing the changes in smoking and drinking status during the 14 year follow-up period according to covariates.
