# Supplementary material for: The Effect of Change of Working Schedule on Health Behaviors: Evidence from the Korea Labor and Income Panel Study (2005–2019)
Source: J Clin Med. 2022 Mar 20;11(6):1725. doi: 10.3390/jcm11061725 (PMC8950370; doi:10.3390/jcm11061725)
Supplement: Supplementary file 1 [file jcm-11-01725-s001.zip › jcm-1603784-supplementary.pdf]

Supplementary Table S1. Results of a generalized estimating equation analyzing the changes in smoking and drinking status during the 14 year follow-up period according to covariates.

Covariates	Healthy to Unhealthy Behavior	Never or Past to Current Smoking	Never or Social to Binge Drinking
	OR (95% CI)	OR (95% CI)	OR (95% CI)
Age (years)			
15~20	1.00	1.00	1.00
21~40	1.04 (0.22-4.82)	0.23 (0.02-2.15)	0.90 (0.19-4.19)
41~60	0.64 (0.14-2.96)	0.14 (0.02-1.37)	0.57 (0.12-2.65)
> 60	0.41 (0.09-1.93)	0.12 (0.01-1.12)	0.35 (0.08-1.64)
Sex			
Male	1.00	1.00	1.00
Female	**0.65 (0.60-0.70)**	**0.07 (0.06-0.09)**	0.95 (0.87-1.03)
Education level			
Middle school	1.00	1.00	1.00
High School	**0.81 (0.74-0.90)**	**0.79 (0.67-0.95)**	**0.82 (0.73-0.92)**
College or higher	**0.72 (0.64-0.81)**	**0.67 (0.55-0.81)**	**0.76 (0.67-0.87)**
Occupational classification			
White-collar	1.00	1.00	1.00
Pink-collar	0.99 (0.89-1.12)	1.09 (0.90-1.34)	0.97 (0.86-1.10)
Green-collar	0.94 (0.46-1.95)	1.14 (0.40-3.23)	0.68 (0.27-1.73)
Blue-collar	0.93 (0.86-1.02)	**0.86 (0.75-0.99)**	0.95 (0.87-1.05)
Monthly wage income ($, USD)			
<1500	1.00	1.00	1.00
1500–2000	0.97 (0.88-1.07)	0.97 (0.80-1.19)	0.96 (0.87-1.07)
2000–2500	1.01 (0.91-1.13)	1.07 (0.87-1.30)	0.99 (0.88-1.12)
>2500	1.03 (0.93-1.15)	**1.25 (1.04-1.52)**	0.93 (0.83-1.05)
Self-rated health status			
Good	1.00	1.00	1.00
Moderate	0.99 (0.92-1.06)	0.96 (0.86-1.08)	0.99 (0.91-1.06)
Bad	1.03 (0.89-1.19)	1.22 (0.96-1.56)	1.02 (0.86-1.20)

Bold indicates statistical significance. Risk is represented as odds ratio (95% confidence interval). All result s were adjusted for age in observation year, sex, education level, occupational classification, monthly wage income level, self-rated health status, and change of working schedule. 'Healthy to unhealthy behavior' ind icates changes from 'Never or past to current smoking' or 'Never or social to binge drinking'.
